# Public Health, Social Medicine and Disease Control: Medical Services, Maternal Care and Sexually Transmitted Diseases in Former Portuguese West Africa (1920–63)

**DOI:** 10.1017/mdh.2018.44

**Published:** 2018-10

**Authors:** Philip J. Havik

**Affiliations:** Clínica Médica, Instituto de Higiene e Medicina Tropical (IHMT), Global Health and Tropical Medicine (GHTM), Universidade Nova de Lisboa, Rua da Junqueira, 100, 1349-008 Lisbon, Portugal

## 1 Introduction

In the immediate post-First World War (WWI) era, growing concerns regarding disease and reproduction in industrialised countries were to result in a marked shift in emphasis towards social welfare and public health. As a result, urban and rural hygiene, housing and nutrition, and the control and eradication of endemic diseases became national and international policy objectives. At the same time, the notion of social hygiene, which was to develop into an ill-defined concept of social medicine, would leave its mark on public health programmes in the interwar period.^1^ After 1945, as the World Health Organization (WHO) redefined the relationship between public health, disease control and social medicine, Cold War politics would, however, obfuscate social determinants in favour of a more epidemiological approach.^2^

Although it largely remained an ‘elusive concept’, social medicine’s basic tenets were associated with a multidisciplinary focus on the social determinants of health in order to counter health inequalities and inform policy, medical practice and research.^3^ Its historical roots go back to mid-nineteenth century ideas on medicine as a social science put forward by in Germany and France by Virchow, Neumann, Leubuscher and Guérin.^4^ Over time, the concept would shift from an emphasis on socio-economic policy reform towards an evidence-based perspective on the correlations between hygiene, health, illness, nutrition, education and housing. Thus, it occupied a transitional field covering public and community health, while manifesting a tension between the dimensions of illness and holistic focus on the person.^5^ The development of social medicine in different political contexts, for example in Europe, North America and Latin America in the 1930s and 1940s, also had an – albeit limited – impact in Africa from the 1940s onwards.^6^ In the interwar years, some national governments and international organisations such as the League of Nations Health Organization and the Rockefeller Foundation’s Health Division were to embrace the notion, which remained strongly associated with the idea of prevention.^7^ As the fields of bacteriology and epidemiology developed and new methods were introduced towards disease control and eradication of endemic diseases, tropical regions under colonial control were transformed into ‘living laboratories’ in search of new scientific tools and ‘magic bullets’.^8^ In European empires in Africa and Asia, ‘biopower’ and the presumed superiority of biomedical technologies were, however, confronted with and often clashed with long standing local socio-cultural perceptions of illness and healing.^9^ These tensions became more visible as hygienist-, medical- and welfare-related concerns informed the organisation of colonial medical and social services in penetrated rural areas where the bulk of ‘indigenous populations’ resided.^10^ Over time, as medical services extended their coverage and vertical campaigns became the rule for combating endemic diseases, above all after 1945, they reached the corners of empires. Thus, indigenous societies increasingly came into contact with large-scale screening, prevention and treatment programmes for a great variety of endemic diseases. At the same time, social medicine began to fill broad lacunas in terms of maternal and neo-natal care, vaccination, nutrition and health education.

The somewhat ambiguous Foucauldian term ‘biopower’ has been the subject of debate regarding its exercise in the context of imperial rule and its racial and gendered dimensions.^11^ However, on the ground the exercise of ‘biopower’ in the sense of changing health-related behaviour was subject to significant barriers as authorities were confronted with local perceptions and agency towards disease and illness. The highly sensitive and culturally embedded issues of sexualisation of female bodies and the pretence exercise of control over their procreative function and social behaviour would be thus associated with a changing mixture of coercive and persuasive discourse and interventions in colonial settings. Within the broader political and administrative framework for population control and engineering, health governance developed a measure of fragmented autonomy that varied not only between but also within colonial territories. In terms of target populations, maternal health and sexually transmitted diseases (STDs) figure as key elements of gendered public health interventions and discourse, which were mainly directed at African women. While notions of economic utility, social hygiene, racial, ethnic and gendered distinctions constituted common dimensions of colonial rule, contrary to the situation in colonial Asia, depopulation anxieties were uppermost in colonial authorities’ minds in Africa. Hence, they would have significant implications for public health and epidemiology, and in particular for policies regarding maternal health and STDs. As the present paper will demonstrate, these were far from static and would change over time to accommodate national and international guidelines and practices. At the same time, the dynamics of personal initiatives by health professionals and local agency were to have a significant impact on medical services, health promotion and prevention.

Whereas British, French and Belgian imperial contexts have benefited from important research inputs, studies on STDs and maternal and child health in colonial Africa have generally overlooked former Portuguese territories. Other than a few studies on Angola,^12^ Mozambique^13^ and Guinea,^14^ the emergence, evolution and use of public health services by Africans remain largely unchartered territory. The present paper provides a fresh comparative perspective on policies and practices regarding STDs and motherhood directed at different local populations in former Portuguese West Africa. It focuses on Portuguese Guinea (PG), current day Guinea-Bissau, while making comparisons with neighbouring French West Africa (Afrique occidentale française, AOF). It aims to fill significant knowledge gaps by means of a longitudinal perspective on health services and epidemiological developments in the territory during a crucial period from the 1920s to the early 1960s, until the outbreak of the colonial war in 1963.

It holds that, despite growing concerns regarding depopulation and contagion, there was a significant gap between policy and practice that only gradually began to narrow in the 1950s. Portuguese health reforms and vertical programmes, and social health and welfare initiatives in neighbouring AOF, which followed WHO guidelines, would be partly responsible for this trend. Far from being homogeneous and linear, however, improvements in human and material resources were markedly uneven in regional and local terms. Two regions, ie. Canchungo and Bafatá, are singled out here for comparison in terms of their impact on maternal health and STD related issues. Policy shifts over time reflected the growing vertical epidemiological approach to STDs and socio-medical concerns with the provision of maternal (and child) care. Available data on STD prevalence indicate that there was a notable imbalance between STD control and maternal and child care. Crucially, personal initiatives by health professionals in the provision of health care would constitute a key factor in the local development of services and changing health seeking behaviour in the 1940s and 1950s. This paper is divided into a comparative section on socio-demographic and public health policies on motherhood and STDs in colonial Africa, followed by two overarching sections on the combat against STDs and the provision of maternal care, respectively, covering the period from 1920 to 1963. The qualitative and quantitative data presented here are largely based upon unpublished administrative and medical reports contained in Portuguese and French archives, as well as on a review of secondary literature.

## 2 Propaganda and Control: Demography, Economy and Public Health

The shift of focus in colonial discourse towards ‘the African family’ from the 1920s took place as medical services and ethnographic surveys began to penetrate the interior, gathering information on local populations and their communities.^15^ In imperial contexts, the perceived African ‘population crisis’ demanded pro-natal policies directed at ‘indigenous’ women, mothers and wives.^16^ These were largely based on European public health programmes for lowering maternal and infant mortality and the control of ‘social’ diseases.^17^ From the early 1900s onwards, famine, disease and epidemics (eg. sleeping sickness, malaria, cholera, yellow fever and bubonic plague) begged urgent sanitary and social improvements.^18^ The ‘demographic panic’ and ‘labour anxieties’ in the interwar years, shared across Africa’s colonial frontiers, were largely based on educated guesses, and unreliable demographic and tax surveys.^19^ Once colonial health services engaged with African populations, a number of barriers and constraints soon became apparent. Socialising medicine implied focusing on the human body and its social, reproductive functions and thus on a group hitherto ignored, ie. women. As a result, maternal/child health and STDs would bring services into the intimate, private sphere while being obliged to increase their effective coverage and improve outcomes, above all for rural populations.^20^

Different threads have been identified with regard to the historiography of maternity in Africa, ie. marriage, education and midwifery on the one hand,^21^ and issues such as contagion, African (female) sexuality and moral order in the case of STDs on the other.^22^ From ‘colonizing the womb’ to ‘making mothers’, and ‘sexual control’ to ‘moral policing’, the literature has largely centred on official narratives with regard to sanitising, modernising and civilising society. But it has also recognised the limits of colonial ‘biopolitics’ and ‘biopower’ as authorities were confronted with challenges posed by their effective implementation.^23^ Political dimensions also played a role, such as the presence of significant settler populations, which tended to accelerate the development of medical, obstetric and child care services,^24^ while racial dimensions of rule also produced considerable differentials in terms of access to services.

Although a lack of human and material resources, infrastructures and know-how^25^ and the impact of the 1929 crisis would slow progress until 1945, thereafter health, welfare and development became official priorities. Interventions in pregnancy, birthing, birth spacing and child rearing posed considerable challenges that were already the subject of debate in Europe and Asia during the nineteenth and twentieth centuries.^26^ However, the burden of failure was often put on the target groups, ie. African societies, owing to their alleged ‘deviant’ social and sexual mores, polygyny, promiscuity, prostitution, ignorance, superstition and lack of hygiene. In addition, religious beliefs, local customs and the role of traditional healers and midwives were seen as obstacles to Africans’ adherence to biomedicine. Although appointed chiefs were expected to collaborate with authorities’ propaganda efforts, locally recruited administrative guards and nurses were often employed as liaisers with local communities.

During the interwar years, the concerns regarding a looming demographic, fertility and ‘family crisis’ was underlined in colonial discourse.^27^ Thus, campaigns for the education of pregnant women in modern notions of hygiene, nutrition and motherhood came to form part of the overall colonial civilising mission and modernisation policies from the 1920s.^28^ As a series of state and civil initiatives ensued to improve maternal and child welfare,^29^ religious missions promoted the moral uplifting of African women as mothers, wives and educators, while combating polygyny.^30^ ‘Social obstetrics’^31^ and auxiliary nursing and midwifery thus emerged as areas of female expertise as limited training facilities became available in colonial capitals.^32^ Although the organisation of maternal and child care for Africans began in colonial Africa in the 1920s and 1930s,^33^ it would only gather momentum after 1945. Medical specialisation and the gradual feminisation of services would provide incentives for the development of health services that were generally the dominion of male professionals.^34^

The rapid spread of STDs such as syphilis and gonorrhoea also invoked racial and gendered narratives, as African, and above all the stigmatisation of women’s, sexuality and alleged promiscuity made them targets of the coercive policing of hygiene and disease.^35^ Beginning with the registration and monitoring of sex workers in towns and the introduction of disease surveillance and registration programmes,^36^ the introduction of new arsenic-based medication for STDs attracted women’s attention.^37^ Assuming allegedly epidemic proportions from the 1920s, colonial governments became overly concerned about the potential impact of syphilis on demography, fertility and morality. Fed by fears of ‘racial degeneration’ and inter-racial sex, the introduction of new diagnostic tests and antibiotics in the 1940s would pave the way for more sophisticated forms of disease control, while providing more accurate and detailed health statistics.

Authorities adopted a dual approach, ie. of persuasion towards African women to mould them into ‘agents of reproduction’, while attempting to curb and control their sexuality as ‘agents of contagion’. Given that African women were seen both as agents of diffusion of disease and as the matrix of new generations, as these initiatives developed, the two threads became increasingly entangled. As the limits of the colonial capacity for control became apparent, awareness of the need for negotiated outcomes grew in medical and administrative circles. The establishment of global benchmarks for health care, service delivery, vaccination, disease control, training and research set by WHO included a strong emphasis on maternal and child health and preventive care.

**Figure 1:**
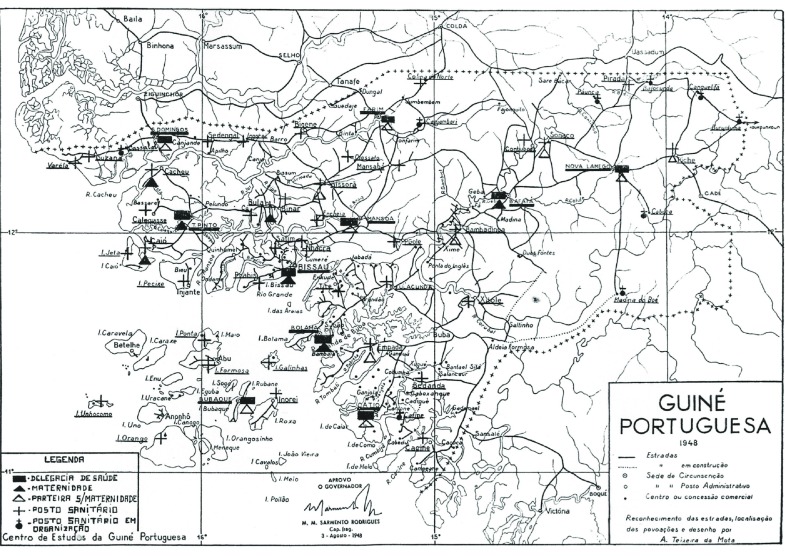
Map of medical facilities, Portuguese Guinea, 1948. Source: A.A. de Barros, Os Serviços de Saúde da Província da Guiné, in: *Anais do Instituto de Medicina Tropical*, XV, 2, 1958: 54.

Similar to other colonial contexts in Africa, health and sanitary legislation and medical services in Portugal’s continental African colonies, Angola, Guinea and Mozambique, would develop from the 1920s onwards to include African populations.^38^ They largely emulated existing models in French and Belgian colonies,^39^ adopting segregated medical facilities for *civilizados* (‘civilised persons’ with the same rights as Portuguese nationals) and *indígenas* (‘natives’, ie. non-citizens).^40^ First targeting ‘natives’ in 1926 in Angola, and thereafter in Mozambique and Guinea, the modest extension of these services to rural areas from the 1930s was based upon the maxim of sanitary assistance being ‘the cornerstone of our colonial settlement’.^41^ Whereas governmental responsibility for public health care was largely alien to metropolitan Portugal under the corporatist New State dictatorship at the time (1926–74),^42^ the state actively engaged in developing these services in empire. Exceptions notwithstanding, the presence and impact of religious missions in social and health care would be significant in Angola and Mozambique, but remained negligible in Portuguese Guinea (PG).^43^ After 1945, health reforms recognised the importance of social medicine and welfare initiatives to reduce the disease burden and mortality rates, increase the birth rate and extend health coverage for African populations in rural areas (Figure 1).^44^

The present paper focuses on PG, a small territory with more than twenty ethnic groups and an estimated total of about 400 000 inhabitants in 1928, when Europeans formed a tiny minority. During the period under consideration, medical services were slow to develop, despite official rhetoric acknowledging the need for expanding care for African populations. State funds for ‘native medical assistance’ created in 1928 and 1933 took due note of the ‘model’ services in neighbouring Senegal under French rule, as well as those developed in Angola from 1926 onwards.^45^ While critical voices rejecting the imposition of a ‘foreign’ blueprint on (deficient) local services saw it as an admission of the ‘poverty of the [colonial] state’, others hailed the pioneering work carried out in Angola as a benchmark for services.^46^

Health services were held to ‘combat infant mortality, increase the birth rate and increase life expectancy’,^47^ while venereal diseases (VD) and prostitution needed to be combated as serious threats to the reproduction of the labour force and to family values.^48^ By the 1950s, some medical personnel began to question the capacity and efficacy of services to implement social medicine, while vertical programmes for the combat of endemic diseases were effectively lowering the prevalence of endemic diseases. The next section will focus first on the combat against STDs, followed by an analysis of policies and practices regarding maternal and child care in Portuguese Guinea for the period from 1925 to 1963, while establishing comparisons with other Portuguese colonial territories and the French AOF.

## 3 Medical Services, Social Order and ‘Native’ Sexuality

From their inception, public health programmes aimed at ‘civilising’ and ‘nationalising’ African communities against the background of demographic, economic and racial concerns. Official colonial discourse highlighted African women’s sexuality, fertility and motherhood roles, while expressing concerns about ‘independent women’ in urban contexts.^49^ Modernisation had unleashed social forces that needed to be brought under control, such as ‘untamed’ female sexuality and the disorder it allegedly caused in society.^50^ Innovations in serological exams with the Wassermann test in 1906, which was used to diagnose syphilis and sleeping sickness, and arsenic-based compounds, ie. first with atoxyl (aminophenyl; arsenic acid) and thereafter with salvarsan (arsfenamine) introduced in 1910, accelerated the process of medicalisation. Two years on a more readily administered compound, neosalvarsan (neoarsfenamine), which soon became available in European hospitals including Portugal, would become the standard treatment.^51^ Thus, the first chemotherapies emerged for both syphilis and Human African Trypanosomiasis (HAT), or sleeping sickness, both regarded as serious demographic threats. Despite their adverse side-effects, these innovations were widely used in mass campaigns devised for HAT and large-scale interventions against STDs.^52^ Once services penetrated rural areas, one of the thorny questions facing policymakers and health professionals was how to effectively reach dispersed and highly mobile African populations.^53^ The alleged predisposition of Africans towards catching and spreading STDs served as a justification for campaigns to control their movements and change their sexual behaviour.

In Portuguese West Africa, the emphasis placed on moralising civil society and curbing sexual promiscuity after WWI can be traced to the early regulation of prostitution by colonial authorities in the late 1800s. Sanitary police corps were set up to control the activities of ‘*mulheres toleradas*’ (‘tolerated women’). Tentative measures to monitor ‘*les filles publiques*’ had already been taken in neighbouring Senegal from the 1860s, as STDs were spreading fast in the capital, St Louis.^54^ In the newly founded capital of PG, Bolama, sex workers who had emigrated from the Cape Verde islands and wandered the town centre were ‘riddled with syphilitic diseases’ and could soon infect the population at large.^55^ Authorities in the commercial capital of Bissau, which had allegedly become a refuge for prostitution and STDs, recommended compulsory inspections.^56^ Women from certain ethnic groups, above all the Manjaco, were singled out from the early 1900s for their physical appearance, hairstyles and dress, and explicitly identified as sex workers (*remadêras* in Guinean Creole (GC), the territory’s lingua franca) with a European clientele in towns such as Bolama and Bissau.^57^ These early references to female sexuality, and to women’s purported responsibility for infecting troops and settlers in the region, provide early glimpses of the colonial state’s gendered discourse and coercive practice directed at African women.

Following the conclusion of military campaigns to subdue the territory’s ethnic groups, authorities issued a new by-law that obliged district administrators ‘to register all women that demonstrably dedicated themselves to prostitution’.^58^ Sanitary police and administrative officials were responsible for ensuring compliance with these regulations and obliged to send those women infected with STDs for clinical treatment. Similar policies had already been implemented in Senegal in 1904 in order to combat clandestine prostitution,^59^ where, by the late 1910s, the first anti-venereal clinic was operating in the *Hôpital Indigène* in Dakar.^60^ Soon, civil society in PG voiced its complaints in the local press, condemning the large-scale evasion by sex workers, the presence of ‘children eaten by syphilis’ and the lack of action by the sanitary police in urban areas.^61^ A decade on, it was evident that the registration system was still ineffective: women who had already registered would fail to show up for regular inspections. Thereupon, authorities opted for awareness campaigns and distributing anti-venereal pamphlets, some versed in ethnic languages, while seeking the cooperation of appointed African authorities.^62^

As public health policies shifted their focus to the interior, they became deeply entangled with demographic, economic and social concerns. Whereas in the mid-1920s, reports identified Creole women as the principal agents of the rapid spread of syphilis, and their ‘mulatto’ progeny were seen as a specific reservoir for malaria and other endemic diseases, by the early 1930s this apparent threat was extended to include a broad array of ethnic communities.^63^ By then, VDs had ‘spread with intensity among the natives’, above all among the matrilineal Bijagó on the homonymous islands – regarded as the least civilised of all groups^64^ – where numbers were ‘frightening’.^65^ The report stigmatised women as the main culprits for the dissemination of this ‘scourge’, which was now seen as ‘a serious threat to the colony’. Similar accusations were directed at women in other colonial contexts, for example with respect to STD outbreaks.^66^ Local health surveys on the islands appear to indicate, however, that the infection rate among Bijagó men was higher than among women.^67^ Similar trends were reported for other areas where the general patient profile centred on ‘black native males’.^68^ Other ethnic groups were also singled out, such as the matrilineal and partly Christianised Manjaco with a high female surplus and known for their considerable mobility and trading skills, while also being regarded as receptive to engaging in sexual relationships with Europeans.^69^ The patrilineal Islamised Fula (Fulbe) were also identified as a high-risk group, owing to their ‘organic deficiency’ and their tendency to omit their medical histories of syphilis, gonorrhoea and chancroid.^70^

The ‘VD momentum’, which extended to the entire colony and further afield to the neighbouring AOF,^71^ was soon regarded as rampant in the interior, threatening the towns and the territory’s economy as a whole: ‘These diseases are attacking the working populations who live around the towns and if nothing is done, [they] will also affect the farming populations.’^72^ Health services observed that patients under treatment for syphilis or gonorrhoea tended to discontinue therapy, and only sought medical advice at a more advanced stage. Alarmed, a critical inspection report stated that ‘syphilis and venereal diseases are strongly contributing to the demise of the population’. A nurse in the ‘deep South’ of the colony found that ‘venereal diseases here are more common than rainwater. Anything goes’.^73^

To counter this threat, the first ‘venereal check-posts’ were set up in the interior in the most affected areas, and some – albeit limited – health promotion was undertaken. Check posts included border regions and insular locations such as the Bijagós archipelago and the Manjaco on the Ilhetas, north of Bissau, while the bush infirmary in Bafatá served the easterly homonymous region.^74^ Reports highlighted the lack of trained personnel: ‘while medical assistance in the [neighbouring] Casamance region [in Senegal] even reached the most remote villages through local auxiliary medical staff, in [Portuguese] Guinea there are no nurses or medication to treat some of the scourges which destroy the native, such as yaws, syphilis and venereal diseases.’^75^ The Manjaco in the Canchungo region (north-west) were said to prefer the ‘modest, but well-equipped hospital in Ziguinchor’ (in the Senegalese Casamance region) to the regional infirmary in their own area.^76^ Inspectors severely criticised the lack of information in reports submitted by native affairs commissioners, which omitted ‘native medical or social assistance’ and failed to monitor local services.^77^ The term *skentamenti* in GC, which does not distinguish between the two diseases, suggested differing local disease taxonomies and perceptions.

From the mid-1940s, medical staff was urged to carry out social surveys in medical districts under their jurisdiction and to study ‘indigenous’ communities from a variety of perspectives, ie. endemic diseases and their aetiology, prophylaxis and vaccines, as well as birth and mortality rates.^78^ These recommendations demonstrated the dearth of health statistics on rural communities, above all those removed from the colony’s road network, and a lack of qualitative data. In urban areas such as Bissau, a raid on sex workers in the 1940s found that 50% of the girls between fifteen and eighteen years of age were carriers of STDs.^79^ Thus, colonial officials advocated the forcible internment and treatment of infected women, while others favoured prophylactic measures.^80^

**Figure 2:**
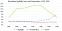
Source: Reports Health Services, 1935–50.

Underreporting of STD cases, owing to the tendency for ‘natives’ to seek assistance for other health conditions, prompted medical staff to gather data on the prevalence of syphilis and gonorrhoea in the interior. The Manjaco and the Fula were singled out for a lack of hygiene and risky sexual behaviour. Hence, the perceived urgency to ‘begin to actively and intensely combat venereal diseases’ seen as ‘social scourges’, especially syphilis. The latter strongly affected the general morbidity and mortality of the population, including children, in the percentage of abortions and stillbirths and the decline of various ‘tribes’ in the colony.^81^ Data show rising prevalence rates of syphilis from 1941 onwards as consultations increased (see Figure 2). They appear to compare favourably to 7,7% prevalence rate in Senegal in 1941, but would soon achieve the similar levels in 1950 (7,8%).^82^

The director of health services alleged that the ignorance of African populations, and above all of women among them, had led the latter to ‘contaminate the population’, thus constituting a serious threat to its survival:^83^

It is difficult to identify the sources of contagion, because the carriers of these diseases hide them, and many are unaware of how they are transmitted, and attribute its appearance to completely ridiculous factors. Manjaco women are those who most spread these diseases, but the few who are found to be sources of contagion and are tracked down, hide and flee, preferably to their home region. Thus, one can understand the problems of eradicating these ailments.

Health officers reported that ‘the intimate contact and promiscuity in which [natives] live with their fellow humans’ in polygynous households was also associated with the dissemination and prevalence of syphilis.^84^ Authorities initiated a campaign against polygyny in the 1940s as part of the ‘civilising mission’, propagating the idea that child marriages fomented adultery and prostitution, lowered the birth rate and increased the number of abortions.^85^ While prostitution was also blamed for its anti-conceptive impact, syphilis was seen as a major cause of spontaneous abortions, together with malaria. Some sources mentioned the demand for phyto-therapeutic contraceptives and abortifacients as well as local pro-fertility medication and curative compounds for STDs.^86^ ‘Sexual promiscuity’ among Africans from an early age was identified as one of the main causes of what was now labelled a ‘social disease’.^87^ Reports also referred to the lack of balanced diets – rich in carbohydrates, but poor in proteins – and the heavy manual labour women performed during pregnancy. As biostatistics on rural populations increased, more cases of congenital syphilis were reported as well as child morbidity and mortality owing to malaria, respiratory diseases, tetanus and tuberculosis.^88^ Medical reports also associated syphilis in its various stages with cardiovascular and mental disorders, suggesting that co-morbidity reduced the effectiveness of the body’s natural defences.

Campaigns intensified from 1945 as new treatments such as penicillin became available as an alternative to sulphonamides, which were increasingly used to treat sick patients, mostly in secondary and tertiary phases. As the venereal disease research laboratory (VDRL) test, in 1942, and the Treponema pallidum immobilisation (TPI) test, developed by Nelson and Mayer in 1949, began to provide greater diagnostic accuracy, reported cases of syphilis increased (see Figure 2), supplemented by increased disease surveillance and newly-built rural sanitary posts.^89^ Some anti-venereal posts had also been built, particularly in certain coastal areas such as the Canchungo region to target mobile populations engaged in coastal shipping and prostitution. As the prohibition of prostitution in 1948 caused it to go ‘underground’, overall prevalence increased: while in 1946, syphilis was still the third most common endemic pathology after malaria, it was second in 1950 with an overall prevalence rate of almost 3%. The situation in rural areas was difficult to ascertain given that nurses’ diagnostics in sanitary posts were omitted from epidemiological data ‘for not being considered valid for statistical purposes’,^90^ while patients would often give other motives for their visit than STDs, thus artificially lowering the clinical register for the latter.^91^

Improved diagnostics also allowed for more specific biostatistics: whereas in 1941, 85% of syphilis cases were undifferentiated, by 1950 these had dropped to just under 30%. During this period, recorded cases of congenital and acquired syphilis showed a six- and an almost seven-fold increase, respectively. Health officials warned, however, that overall syphilis prevalence was seriously underestimated. In some areas in the interior, such as the Bafatá region where modern tests and chemotherapy were not always available to distinguish different treponemes, syphilis was increasing ‘day by day’; however, cases of yaws had diminished as patients sought treatment.^92^ But, by the late 1950s, no significant reduction in prevalence had yet been achieved in the Bafatá region.^93^ WHO guidelines and support programmes linked the combat of STDs to maternal and child care.^94^ Anti-venereal campaigns were partially implemented, albeit unevenly. Nevertheless, improvements in health and social services were slow to materialise owing to a lack of funding and personnel. An example of cross-border cooperation recommended by WHO, was the mass screening and treatment of yaws implemented simultaneously in Senegal and GP in the mid-1950s.

**Figure 3:**
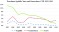
Source: Annual Reports MCDESE, 1953–62.

These campaigns signalled a policy shift towards vertical campaigns. In PG, they were now coordinated by the Commission for the Combat against Sleeping Sickness and Endemic Diseases (MCDSE), established in 1945. MCDSE initiated a broad mass screening programme in rural areas in 1952, similar to the *Service Général d’Hygiène Mobile et de Prophylaxie* (SGHMP) in French Africa from 1944–45. Employing nine full-time tropical medical experts by 1958, following WHO guidelines and with its own facilities and mobile teams, MCDSE filled considerable gaps left by regular services in rural areas. Detecting hitherto undetected cases of yaws, syphilis and gonorrhoea, it significantly reduced their prevalence. However, gonorrhoea prevalence appeared to be increasing by the late 1950s (see Figure 3), a trend which would continue into the next decade.^95^

## 4 Public Health and African Motherhood

In the early 1920s the French consul in the commercial capital and port of Bissau found that health services there were minimal owing to a lack of personnel, ‘there was only one MD, and no pharmacist, nurse or midwife’.^96^ His observations laid bare problems that the neighbouring colony of Senegal had been dealing with from the early 1900s, ie. to increase the coverage of clinical and mobile services in remote rural areas. The emergence of the *assistance medicate indigene* (AMI) in the AOF in 1905, and the establishment of a course for ‘native’ midwives in 1922 at the Medical School in Dakar, set important examples for the West African region. Justified by demographic anxieties, the introduction of the PMI (*Protection Maternelle et Infantile*) policy, first in France and thereafter in its colonies after WWI, inspired the building of maternity facilities directed at African women. AMI mobile teams, and later those pertaining to the SGHMP roaming the interior, identified pregnant women and attempted to persuade them to take gynaecological exams and accept social obstetrics.^97^ Contemporary debates in French and British West Africa reveal the reduction of maternal and infant mortality rates and the care provision that emerged as overriding issues after WWI.^98^ Budget priorities and a lack of funding provoked delays in the implementation of government measures in the Gold Coast, including maternity clinics and training facilities for midwives, in the early 1920s.^99^ In Nigeria, the first measures towards creating maternity clinics were introduced in the mid-1920s – in conjunction with facilities to treat STDs – as well as infant health care and education for young mothers on hygiene and nutrition.^100^

In the 1920s, infant and adult mortality and morbidity rates, malnutrition and social change began to be viewed as serious threats to African populations’ survival in Portuguese colonies. At the First West African Conference on Tropical Medicine, held in Angola in 1923, the ground-rules were established for preventive sanitary campaigns focusing on Africans in Portuguese colonies. An excessive focus on urban centres had left African communities in the interior practically bereft of medical assistance. Thus, ‘the native’ needed to be targeted with mobile services, while medical staff was expected to act both as clinical and social agents.^101^ PG would follow developments in Angola, albeit with some delay. The Fund for Native Medical Assistance (1932), meant to fill this conspicuous lacuna, was inspired by a similar service initiated in Angola in 1927.^102^ These public funds were also inspired by a public health programme in neighbouring Senegal, which provided medical extension to rural areas to reduce infant mortality.^103^ While these services were said to be free of charge, the preamble of the decree admitted that services were underfunded and incapable of reaching their principal target, ie. the ‘native’, in all the corners of the colony.^104^ Thus, fiscal surcharges on the hut tax constituted the main financial source for the Fund. Village or rural infirmaries, or *tabanca*-*enfermarias* – known as *enfermarias-senzala* in Angola – were solely directed at ‘native Africans’ and organically separate from existing medical services, whose personnel were expected to contribute without additional remuneration.^105^ Given that Africans faced approximately eight months of illness a year, on a population of over 400 000 inhabitants, services employing only seven medical doctors and twenty-seven nurses were ill equipped to cope with their needs (see Figure 5). The Franciscan Catholic Mission, established in the colony in 1931, would also receive funding from the aforesaid Fund for educational and medical purposes, but they had limited impact.^106^

**Figure 4:**
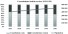
Source: Reports Health Services, 1933–50.

A report based on the 1934 ethnographic survey enunciated the notion of African family life, providing extensive information on local sexual-, marriage-, pregnancy- and birthing customs and rites of passage.^107^ Other official reports underlined the lack of adequate care given by ‘native mothers’ to their offspring, thus provoking (unnecessary) illness and causing (untimely) deaths.^108^ Thus, rudimentary village infirmaries – the first being built in the south of the colony in Fulacunda and in the east in Bafatá in 1933/4 – equipped with ‘native-like huts’, were meant to attract African communities and provide maternal and neo-natal care. Whereas women increasingly sought assistance (see Figure 4), they did not necessarily seek ante-natal, natal or child care services, but rather treatment for certain endemic diseases such as yaws, syphilis and gonorrhoea.^109^

Although awareness campaigns appeared to be having some effect, Catholic missionaries criticised state infirmaries and dispensaries for their lack of organised care. They quoted ‘natives’ praising the services rendered in the neighbouring AOF, while declining to seek assistance from local sanitary posts.^110^ A boarding school for orphans erected by Catholic missionaries close to Bissau in 1933, where nurses ran a kindergarten and gave education to girls and future mothers,^111^ was dismissed by an administrative inspector as a ‘school for housewives and child-minding’.^112^ Official reports began to recognise the need for modern maternity wards, kindergartens and dispensaries for ‘native mothers’, where they would receive instructions on hygiene and therapeutic care ‘as tools for protecting [natives] against the outward signs of physical degeneration…to strengthen racial robustness’.^113^ They were meant to respond to local communities’ needs by improving medical assistance and medicalisation in sanitary posts and village infirmaries.^114^ However, by the early 1940s, only two hospitals in PG, in Bissau and Bolama, had maternity wards, with few beds for African women, while construction work on new units was still underway.^115^ Although statistics for assisted delivery in the colony’s few hospitals confirm the limited recourse of ‘indigenous’ women to these services, they also provide early records of intra-uterine deaths, septicaemia, childbirth accidents, ovarian and genital/urinary conditions in the twenty–thirty-nine age bracket.^116^

Following a critical inspection report, the head of medical services squarely laid the blame on traditional midwives for lacking basic skills, sometimes resulting in the death of the child and/or the mother; spontaneous and induced abortions and miscarriages were common. Unspecified infant mortality levels remained ‘alarming’ and allegedly caused by African mothers’ ignorance of post-natal care, breastfeeding, nutrition and hygiene. Nevertheless, authorities warned against forcing women to seek assistance. Legislative reforms in empire in 1945 recognised the need for the health education of pregnant women, provision of maternal and child care and the training of local nurses, midwives and *visitadoras* (visiting nurses).^117^ In neighbouring Senegal and French Guinea, where African women had shown little enthusiasm for maternity clinics, in the mid-1930s authorities had already opted for home-assisted births with the aid of locally-trained nurse-midwives, with encouraging results.^118^ In PG, reports also acknowledged the lack of post-natal care as a major hindrance, whereas the most elementary paediatric norms were largely ignored. Although mothers were observed for a few days post-partum, their new-born were not: weight and breastfeeding were not monitored regularly. In some district capitals (ie. Bolama, Canchungo and Cacheu), maternity wards were not operational, while some sanitary posts were effectively closed owing to absence of personnel, equipment or medication.^119^ These reports also criticised the dearth of maternity wards and infirmaries in regional medical centres and the constant delays in construction work.^120^

**Figure 5:**
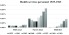
Source: Reports Health Services, 1925–63.

**Figure 6:**
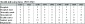
Source: Reports Health Services, 1925–63.

Visits to villages now identified insufficient and unbalanced nutrition as the main cause of infant mortality, while some cases of congenital syphilis were reported. Its low prevalence suggests that symptoms such as bone deformities, dermatological and intraocular lesions may not have been diagnosed as being syphilis-related at the time. A campaign to build – mostly rudimentary – sanitary facilities in the interior was put in place from 1946 onwards, this time funded through a loan from Lisbon. Authorities’ aimed to provide ‘a room in each (small) permanent health post to attract native women to deliver their children, assisted by a nurse and a medical doctor…Money will not be in short supply’.^121^ While the then governor held that services in PG were equal to those in the AOF, critics argued that they ‘looked perfect on paper, but not in practice’.^122^ The patent lack of medical doctors (one per 50 000), nurses (one per 14 000) and trained midwives (until 1946 there was only one for the whole colony) illustrated the low priority given to human resources and medical infrastructures (see Figures 5 and 6). In addition, health professionals were largely male: in 1946, only 10% of nurses were female. A course for auxiliary midwives was inaugurated at the Bissau Hospital in 1948; they were to assist district health officers in ‘their social and humanitarian tasks’. However, the initial results were disappointing, given the limited availability of medical personnel.^123^

In 1950, the principal maternity ward in the capital, Bissau, handled an average of fifteen births a month for a city of over 17 000 inhabitants, 80% of which were African.^124^ In the district capital, Canchungo, (1892 inhabitants, 90% ‘natives’), where the infirmary had only one room with three beds, twenty-two births were recorded in the ward between 1955–57, while seventeen were born at home. In the small town of Cacheu, only four assisted births occurred in the infirmary and eleven locally.^125^ In the Bafatá region only 127 births were reported during the same period on a population of 67 370 inhabitants.^126^ While these numbers are very low, certainly compared to local demographics, they showed that medical services in some areas had begun to assist homebirths. Importantly, reports also show that these initiatives were largely personal. When individual MDs or nurses saved the life of a pregnant mother and her child, their local prestige would increase and, as a result, acceptance of medicalisation.^127^ In the 1940s, when the first maternity ward was inaugurated in the Canchungo infirmary, the only certified (Portuguese) midwife in the colony started a course for ‘*visitadoras*’ to visit villages and assist nurses in their domestic visits to pregnant women (GC: *preñada*). However, this initiative was not consensual; while the governor supported it, the then director of health services rejected the idea.^128^ Nevertheless, his successor did decide to train local nurses in midwifery, while district health officers advocated the establishment of small, regional maternity infirmaries, paediatric, obstetric and gynaecological services and maternal/child-care centres. They argued that their absence was: ‘extremely damaging to the care for native women giving birth and for their offspring. We are convinced that the existing medical assistance for homebirths does not satisfy the sanitary goals that have been set.’^129^

Officials admitted that women continued to show great reluctance towards gynaecological consultations, although more local mothers-to-be were attracted to maternity wards as they were being built in the interior.^130^ Some reports explained Guinean women’s lack of enthusiasm as related to their preference for giving birth at home in a squatting position (GC: *padi*) assisted by their *mamanas* (GC: traditional midwives).^131^ Other officials noted that women and children often missed mass screening and vaccination campaigns because they were working in the fields.^132^ But again, services did not further investigate these issues. Facilities for lactating women and their offspring were planned, to be run by Franciscan nuns, suggesting that earlier tensions between the state and the Church were being resolved after the signing of the 1940 Concordat. However, by the late 1950s these services were not yet operational and missionary inputs remained limited to a few nurses working in state hospitals.

Antenatal consultations were only sought by a few ‘civilised’ women, which meant that MDs were only attending pregnant women with complications. ‘Native’ women were hard to convince to seek medical advice ‘when they were feeling well’, only bringing along their children ‘when they were really sick or in a serious condition which leaves us with few options to assist them’.^133^ On some occasions, when pregnant women died in childbirth and the progeny survived, medics acted as godparents.^134^ In the 1950s, as infirmaries for women began to be built in rural areas, they appeared to react favourably, preferring them to maternity wards in hospitals. The empathy and caring attitudes shown by individual nursing staff and midwives in some infirmaries, for example in Canchungo, also attracted local patients for deliveries.^135^ Rather than employing propaganda, some health officials began personally ‘negotiating’ care outcomes in ‘utilitarian’ clinics providing services tailored to local needs. Not surprisingly, facilities in the Canchungo region would become the best equipped and attain greater regional coverage: in the early 1960s, six of the fifteen rural maternity infirmaries in the colony were located there. Meanwhile, health officers in other districts, such as Gabú, Farim, São Domingos, Fulacunda, Buba and Catió (see Figure 5), still complained about the lack of midwives, and lack of maternity and transport facilities (eg. bicycles) for midwives which kept them from visiting pregnant women and provide counselling on hygiene and child care.^136^ Although assisted births show a more than seven-fold increase between 1950 and 1963, in nominal terms the numbers remained very low (2654 in 1963) compared to the overall female population of fertile age.

## 5 Final Considerations

This paper focused on a crucial time-frame for the establishment and growth of health services in colonial Africa. From the 1920s, these services expanded significantly in British, French and Belgian colonies, mainly on account of depopulation anxieties. Their growth after 1945 in former Portuguese colonies, and the advocacy of social medicine, suggested improvements for African populations as- demographic concerns diminished. However, as the paper has shown, progress was slow and uneven in colonial contexts, while local responses to these services were mixed and selective. A dual approach towards STD control and maternal health emerges here: ie. a repressive one towards African women depicted as sex workers and disseminators of disease, and a paternalist discourse reserved for women as (purportedly inept) progenitors of future generations. Evidence suggests that while coercive measures towards women were preferred regarding STDs, a more persuasive approach was favoured for medicalising maternal health and birthing. Nevertheless, coercive interventions would sometimes undermine public health efforts: the prohibition of prostitution decreed in 1948 actually countered disease control for this high-risk group of women. Racial legislation and policies would also contribute to the belated shift towards the medicalisation of childbirth and neo-natal care for Africans, markedly contrasting with stated public health policies as well as developments in neighbouring colonies such as Senegal and the AOF.

The data presented above illustrate certain trends for the period under consideration in terms of the combat against STDs and the provision of maternal care in Portuguese West Africa, and PG in particular. First, despite the shift in discourse towards public health and epidemiological control in the 1920s, primary care for African populations remained very limited, owing to political priorities, funding, human resources, facilities and racial segregation in health. Contrary to colonial trends, the input of religious missions remained modest. Health reforms in empire in 1945 appear to signal a policy shift towards social medicine, preventive action and health education, aided by improved diagnostics and the use of antibiotics. Until the early 1940s, health services in Angola, Mozambique and Guinea only had one midwife each among their ranks. In PG, staffing levels, including medics, nurses and midwives, largely remained unaltered until the late 1940s despite a ten-fold increase of patients between 1924 and 1948. A three-fold increase in nurse-midwives and a 75% increase in nurses between 1948 and 1960 still left health facilities in most rural health areas seriously under-resourced.

Secondly, regional factors played an important role in these changes, above all public health and welfare programmes introduced in neighbouring Senegal, French Guinea and the AOF. Although Portuguese campaigns against STDs, yaws and tuberculosis would largely attempt to emulate their French neighbours, the fragile health services in PG proved incapable of implementing them, just like those in Angola.^137^ As regular services in PG recorded an increase in syphilis prevalence from the early 1940s, as well as of malaria and tuberculosis, a vertical approach was implemented by the Commission for the Combat Sleeping Sickness and other Endemic Diseases. Unlike Angola and Mozambique, it covered PG as a whole, working with fixed and mobile networks, effectively reducing the prevalence of a range of endemic diseases over the next decades. Whereas prevalence of HAT, syphilis and yaws diminished, gonorrhoea began causing some concern from the early 1960s. Nevertheless, regular services remained responsible for (limited) socio-medical care. While maternal- and child health and social care in neighbouring Senegal made significant advances, health services in PG were slow to implement them. Only the outbreak of the colonial war in 1963 would serve to accelerate investments in public health and welfare projects, including maternal and child health.^138^

Thirdly, in a broader international context, international organisations such as the WHO would be instrumental in encouraging inter-African cooperation in health resources and epidemiological surveillance and control. The newly created Regional Office for Africa (AFRO), led by a Portuguese epidemiologist from 1954 until 1964, introduced vertical as well as inter-regional programmes for combating tuberculosis, yaws and STDs, while promoting the use of more accurate diagnostic tests and new treatments.^139^ It also proposed improvements in maternal and child health to reduce maternal and infant mortality and morbidity, providing multilateral funding for training and internships in public health, maternal and child health, nutrition and health education. However, in the case of Portugal’s African territories, the bulk of training grants were attributed to staff in Angola and Mozambique, and only very few to health personnel in PG. Sometimes, the latter were obliged to withdraw their candidacy owing to staff shortages.^140^ A survey proposed by AFRO in the mid-1950s, on the combat against venereal and treponemal diseases, received a negative response from health authorities in PG, reporting that physicians failed to show interest.^141^ Well-trained staff working with vertical MCDSE campaigns coordinated by the Portuguese Institute of Tropical Medicine (IMT) in Lisbon did however benefit from support provided by international networks such as the WHO. Following WHO expert committee guidelines, they introduced fixed and mobile mass screening, research and treatment programmes in rural areas, filling gaps left by regular services and significantly reducing the prevalence of endemic diseases, including STDs.

Fourthly, the development of services and their outcomes would be uneven within the colony. As medical reports became more detailed in the 1940s, they revealed significant regional differences. Two areas were singled out: the Canchungo and Bafatá regions, characterised by particularly mobile and economically active populations (Fula and Manjaco) with a high prevalence of infectious diseases, including yaws, syphilis and gonorrhoea. The Canchungo region, which accounted for almost 50% of all yaws cases in the colony in 1941 and 1945, would become a laboratory for policies aimed at STDs as well as for maternal care. The region’s main ethnic group, the highly mobile Manjaco, were regarded as key contributors to the Guinean economy, but also heavily stigmatised – above all, the women among them – as a high-risk group.^142^ At the same time, the Bafatá region remained heavily under-resourced, with ill-adapted facilities and a lack of qualified personnel. The impact of social obstetrics and medicalisation was therefore uneven, as certain areas and populations were targeted while others lagged behind. When anti-venereal posts and a maternal/child-care centre were opened in Canchungo in the 1950s,^143^ these examples were not necessarily followed elsewhere.

Fifth, while direct references to the role of populations in shaping medical services and responses remain limited and ethnographic research on health-related subjects was rare, administrative and health reports do provide some glimpses of these local dynamics. Fragmented reporting on traditional African perceptions of illness, medical services and health promotion campaigns suggest that pluralist medical strategies were common, and that health-seeking behaviour was a negotiated process. Local demand for services was highly selective, motivated by the perceived efficacy of treatment in the case of yaws and syphilis and the preference for homebirths. As small maternity infirmaries served by auxiliary midwives were built from the late 1950s adjacent to sanitary posts in the Canchungo region, women’s adherence was said to be ‘remarkable’,^144^ but the flux of female patients to village infirmaries shows that their inclusion in patients’ trajectories was not only selective but also surreptitious in the case of sexually related ailments, owing to stigma, and did not necessarily imply local acceptance of their suitability for deliveries, maternal or infant care. Oral data convey health professionals’ lack of or very limited knowledge of GC or ethnic vernacular, as well as of local perceptions of illness and birthing, largely relying on auxiliary personnel for (cultural) translation.^145^

Finally, evidence gathered from health reports and oral data show that personal inputs by colonial health professionals functioned as key drivers for change. These initiatives – which remain under-researched in colonial contexts – appear to have been directed at maternal care rather than the treatment of STDs. More than colonial ‘biopolitics’, personal engagement by female nurses and midwives, and a few male health professionals, created innovative – albeit localised – dynamics, which shaped culturally sensitive socio-medical care. By the late 1950s, health officers in PG still complained that maternal care for Africans largely depended on health professionals’ personal initiative as they coped with the chronic lack of resources, lamenting the inefficacy of services. The increase in local female auxiliary staff and the collaboration of female MDs – which was prohibited until the mid-1950s^146^ – may also have contributed to a greater afflux of African women in certain areas. The gradual feminisation and Africanisation of services, above all at auxiliary level, and its potential impact on health seeking behaviour and service delivery during the 1960s and early 1970s, unfortunately go beyond the limits of this paper.

## Footnotes

1.
Iris Borowy, ‘International Social Medicine between the Wars: Positioning a Volatile Concept’, *Hygiea International*, 6, 2 (2007), 13–35.

2.
Randall M. Packard, *A History of Global Health: Interventions into the Lives of Other Peoples* (Baltimore: Johns Hopkins University Press, 2016), 105–131.

3.
Dorothy Porter, ‘How Did Social Medicine Evolve and Where is it Heading?’, *PloS Medicine*, 3, 10 (2006), e399.10.1371/journal.pmed.0030399PMC162109217076552

4.
George Rosen, ‘Social Medicine: A Genetic Analysis of the Concept’, *Bulletin of the History of Medicine*, 21, 5 (1947), 674–733.18901361

5.
Ute Gerhardt, ‘The dilemma of social pathology’, in Dorothy Porter, *Social Medicine and Medical Sociology in the Twentieth Century* (Amsterdam: Rodopi, 1997), 137–64.

6.
On the impact of social medicine in Latin America, see Howard Waitzkin, *Medicine and Public Health at the End of Empire* (Abingdon/New York: Routledge, 2016), 156–70; for Africa, see Shula Marks, ‘South Africa’s Early Experiment with Social Medicine: Its Pioneers and Politics’, *American Journal of Health*, 87, 3 (1997), 452–9.10.2105/ajph.87.3.452PMC13810249096553

7.
Porter, *op. cit.* (note 3), and Borowy, *op. cit.* (note 1).

8.
Helen Tilley. *Africa as a Living Laboratory: Empire, Development and the Problem of Scientific Knowledge* (Chicago/London: University of Chicago Press, 2011), 169–216.

9.
Steven Feierman, ‘Struggles for Control: The Social Roots of Health and Healing in Modern Africa’, *African Studies Review*, 28, 2/3 (1985), 73–147; Megan Vaughan, ‘Healing and Curing: Issues in the Social History and Anthropology of Medicine in Africa’, *Social History of Medicine*, 7, 2 (1994), 283–95.11616461

10.
Ulrike Lindner, ‘The Transfer of European Social Policy Concepts to Colonial Africa: The Case of Maternal and Child Welfare’, *Journal of Global History*, 9, 2 (2014), 208–231.

11.
See Ann Laura Stoler, *Carnal Knowledge and Imperial Power: Race and the Intimate in Colonial Rule* (Berkeley: University of California Press, 2002). Taking its cue from an ill-defined notion of biopower originally proposed by Foucault, a distinction between ‘hard’ and ‘soft’ biopower has been proposed, referring to forms of bio-social engineering which focus on the individual body and its performance on the one hand, and on population health and regulatory control on the other. See Paul Rabinow and Nikolas Rose, ‘Biopower Today’, *Biosocieties*, 1 (2006), 195–217.

12.
Samuel Coghe, ‘Medical demography in interwar Angola: measuring and negotiating health, reproduction and difference’, in A. Widmer and V. Lipphardt (eds), *Health and Difference: Rendering Human Variation in Colonial Engagements* (New York: Berghahn Books, 2015), 178–214, and also by same author, ‘Inter-imperial Learning and African Health Care in Portuguese Angola in the Interwar Period’, *Social History of Medicine*, 28, 1 (2014), 134–55.

13.
Valdemir Zamparoni, ‘Leprosy: Disease, Isolation and Segregation in Colonial Mozambique’, *História, Ciências, Saúde- Manguinhos*, 224, 1 (2014), 13–39; Francis Dube, ‘Colonialism, Cross-border Movements, and Epidemiology: A History of Public Health in the Manica Region of Central Mozambique and Eastern Zimbabwe and the African Response, 1890–1980’ (unpublished PhD dissertation: University of Iowa, 2009).10.1590/S0104-5970201600500002827849217

14.
Philip J. Havik, ‘Public Health and Tropical Modernity: The Combat Against Sleeping Sickness in Portuguese Guinea (1945–74)’, *Manguinhos: história, ciências, saúde*, 21, 2 (2014), 641–66.10.1590/s0104-5970201400500001325055331

15.
Philip J. Havik, ‘Les Noirs et les “Blancs” de l’Ethnographie Coloniale: discours sur le genre en Guinée Portugaise, (1915–35)’, *Revue Lusotopie*, XII, 1–2 (2005), 55–76.

16.
Carol Summers,‘’ Intimate Colonialism: The Imperial Production of Reproduction in Uganda, 1907–25’, *Signs*, 16, 4 (1991), 787–807: 788.

17.
Lindner, *op. cit.* (note 10), 212–15.

18.
Jill Dias, ‘Famine and Disease in the History of Angola, *c.*1830–1930’, *Journal of African History*, 22, 3 (1981), 349–78.10.1017/s002185370001957511632222

19.
Samuel Coghe, ‘Tensions of Colonial Demography: Depopulation Anxieties and Population Statistics in interwar Angola’, *Contemporânea*, 18, 3 (2015), 472–78.

20.
Summers, *op. cit.* (note 16), 807.

21.
Anne Hugon, ‘L’historiographie de la maternité en Afrique subsaharienne’, *Clio*, 21 (2005), 212–29: 4.

22.
Vaughan, *Curing Their Ills: Colonial Power and African Illness* (Cambridge: Polity Press, 2004), 149–52.

23.
Vaughan, *ibid.*, 202; Nancy Rose Hunt, ‘STDs, suffering, and their derivatives in Congo-Zaire: notes towards an historical ethnography of disease’, in: C. Becker, J.-P. Dozon, C. Obbo and M. Touré M. (eds), *Vivre et Penser Le Sida en Afrique* (Paris: Karthala, 1999): 111–31; Nancy Rose Hunt, *A Colonial Lexicon of Birth Ritual, Medicalization and Mobility in the Congo* (Durham and London: Duke University Press, 1999), 237–80.

24.
Lindner, *op. cit.* (note 10), 223–29.

25.

*Ibid*.

26.
For a general appraisal, see Hilary Marland and Anne Marie Rafferty (eds), *Midwives, Societies and Childbirth: Debates and Controversies in the Modern Period* (New York: Routledge, 1997). On Portugal, see Renilda Barreto, ‘A ciência de parto nos manuais portugueses de obstetrícia’, *Niterói*, 7, 2 (2007), 219–36. For Asian contexts, see Sara Hodges (ed.), *Reproductive Health in India: History, Politics, Controversies* (New Delhi: Orient Longman, 2006).

27.
Margaret Lock and Vinh-Kim Nguyen, *An Anthropology of Biomedicine* (Oxford: Wiley-Blackwell, 2010), 157–58; Vaughan, *op. cit.* (note 22), 130; Shula Marks and Richard Rathbone, ‘The History of the Family in Africa: Introduction’, *Journal of African History*, 24, 2 (1983), 145–61.

28.
Meredith Turshen, ‘Reproducing labour: colonial government regulation of African women’s reproductive lives’, in Karl Ittmann, Dennis D. Cordell and Gregory H. Maddox (eds), *The Demographics of Empire: The Colonial Order and the Creation of Knowledge* (Athens: Ohio University Press, 2010), 217–44.

29.
Hunt, *op. cit.* (note 23), 237–80.

30.
Andrew E. Barnes, “‘Making Good Wives and Mothers”: The African Education Group and Missionary Reactions to the Phelps Stokes Reports’, *Studies in World Christianity*, 21, 1 (2015), 66–85.

31.
Hunt, *op. cit.* (note 23), 250–52.

32.
Nana A. Amponsah, ‘Colonizing the Womb: Women, Midwifery, and the State in Colonial Ghana’ (PhD dissertation: University of Texas, 2011), 225. See also, Pascal Barthélemy, *Africaines et diplômées à l’époque coloniale (1918–57)* (Rennes: Presses Universitaires de Rennes, 2010); Pascal Barthélemy, ‘Sages-femmes africaines diplômées en AOF des années 1920 aux années 1960’, in Anne Hugon, *Histoire des Femmes en Situation Coloniale: Afrique, Asie, XXe siècle* (Paris: Karthala, 2004), 119–44; Sheryl Nestel, ‘(Ad)ministering Angels: Colonial Nursing and the extension of empire in Africa’, *Journal of Medical Humanities*, 19, 4 (1998), 257–77.10.1023/a:102490811002111620458

33.
Lindner, *op. cit.* (note 10); Amponsah, *ibid.*; Deanne van Tol, ‘Mothers, Babies, and the Colonial State: The Introduction of Maternal and Infant Welfare Services in Nigeria, 1925–45’, *Spontaneous Generations* 1, (2007); Hugon, *op. cit.* (note 21)); Lynn M. Thomas, *Politics of the Womb: Women, Reproduction and the State in Kenya* (Berkeley: University of California Press, 2003); Jean Allman, ‘Making Mothers: Missionaries, Medical Officers and Women’s Work in Colonial Asante, 1924–45’, *History Workshop*, 38 (1994), 23–47; Summers, *op. cit.* (note 16).10.1093/hwj/38.1.2311639388

34.
Lindner, *op. cit.* (note 10), 228–29; Hunt, *op. cit.* (note 23), 130, 200.

35.
Lindner, *op. cit.* (note 10), 223–29.

36.
Daniel J. Walther, *Sex and Control: Venereal Disease, Colonial Physicians and Indigenous Agency in German Colonialism, 1884–1914* (New York: Berghahn, 2015); Mor Ndao, ‘Les prises en charge des pathologies vénériennes par le pouvoir colonial au Sénégal, 1850–1960: éclairages et enseignements pour le sida’, *Annales de la Faculté des Lettres et Sciences Humaines* (Dakar), 39, 2011, 41–67; Idrissou Alioum, ‘Prostitution et lutte contre les maladies vénériennes au Cameroun, 1923–64: discours et pratiques’, *Kaliao*, 2, 4 (2010), 123–41; Vaughan, *op. cit.* (note 22), 129–54 ; Bryan T. Callahan, *Syphilis and Civilization: A Social and Cultural History of Sexually Transmitted Disease in Colonial Zambia and Zimbabwe* (Baltimore: Johns Hopkins University, 2002); Karen Jochelson, *The Colour of Disease: Syphilis and Racism in SA, 1880–1950* (New York/Oxford: Palgrave/St Anthony’s, 2001); Michael W. Tuck, ‘Syphilis, Sexuality and Social Control: A History of Venereal Disease in Colonial Uganda’ (PhD thesis: NorthWestern University, 1997).

37.
See Vaughan, *op. cit.* (note 22), 150–51.

38.
See ‘Regulamento Geral dos Serviços de Saúde da Província de Moçambique, Portaria 1480’, *Boletim Oficial de Moçambique*, 10 April 1920; ‘Regulamento Geral dos Serviços de Saúde da Província de Angola, Portaria 102’, *Boletim Oficial de Angola*, 15 April 1920; ‘Regulamento Geral dos Serviços de Saúde da Província da Guiné Portuguesa’, *Boletim Oficial da Guiné Portuguesa*, 10 April 1920.

39.
Coghe, *op. cit.* (note 12), 181.

40.
Philip J. Havik, ‘Saúde pública, microbiologia e a experiência colonial: o combate à malária na África Ocidental (1850–1915)’, in Renilda Barreto and Cristiana Bastos (eds), *A Circulação do Conhecimento: medicina, redes e império* (Lisbon: Instituto de Ciências Sociais, 2013), 317–50.

41.
Armindo Monteiro, ‘Sessão Inaugural da I Conferência do império’, in: *Para uma política imperial: alguns discursos do Ministro das Colónias* (Lisbon: Agência Geral das Colónias, 1933).

42.
Rui Manuel Pinto Costa, *O Poder Médico no*Estado Novo *(1945–74)*: *afirmação, legitimação e ordenamento profissional* (Oporto: Universidade do Porto, 2009).

43.
Coghe, *op. cit.* (note 12), 180–81.

44.
Decree no. 45541, ‘Regulamento dos Serviços de Saúde e Assistência do Ultramar’, 23 January 1945 (Lisbon: Agência Geral do Ultramar, 1945).

45.
Acta do Conselho de Saúde e de Higiene Pública, Bolama, 8 July 1926; AHU (Arquivo Histórico Ultramarino), MU (Ministério do Ultramar), DGAPC/RSH, 1920–6; Philip J. Havik, ‘’From Hospitals to Villages: The Organisation of Health Services, Disease Control and Social Medicine in Portuguese West Africa’, *Portuguese Studies Review*, 25, 2 (2017), 17–56.

46.
António Leite de Magalhães, ‘Relatório sobre o mandato 1927–30’, Lisbon, 31 July 1931; AHU, MU, DGCOcd 417; Eurico de Almeida, *Da assistência médica aos indígenas na Colónia da Guiné Portuguesa* (Bolama: Imprensa Nacional, 1935), 16.

47.
A. Damas Mora, ‘A mortalidade infantil de brancos e indígenas nas colónias de Angola e Moçambique, suas causas principais e remédios possíveis’, *Africa Médica*, VII, 5 (1941), 137–54.

48.
J. da Silva Neves, ‘A luta anti-venérea nas colónias’, *Revista Medica de Angola*, 3, 4 (1923), 169–77.

49.
Sheryl McCurdy, ‘Urban threats: Manyema women, low fertility and venereal diseases in Tanganyika, 1926–36’, in Dorothy L. Hodgson and Sheryl A. McCurdy (eds), *Wicked Women and the Reconfiguration of Gender in Africa* (Portsmouth/Oxford/Cape Town: Heinemann/James Currey/ David Philip, 2001), 212–33.

50.
Vaughan, *op. cit.* (note 22), 131; Walther, *op. cit.* (note 36), 13–14.

51.
Cristiana Bastos, ‘Thomas de Mello Breyner e a clínica da sífilis’, in Cristiana Bastos (ed.) *Clínica, Arte e Sociedade: a sífilis no Hospital do Desterro e na saúde pública* (Lisbon: Imprensa de Ciências Sociais, 2011), 163–73.

52.
Summers, *op. cit.* (note 16), 790–92.

53.
Jochelson, *op. cit.* (note 36), 142–43.

54.
Kalala J. Ngalamulume, ‘Le péril venérien: l’État colonial français et la sexualité à St Louis du Sénégal, 1850–1920’, in Jean Paul Bado, *Les Conquêtes da la Médicine Moderne en Afrique* (Paris: Karthala, 2006), 99–118.

55.
Pedro Ignacio de Gouveia, *Relatório do Governador da Província da Guiné Portuguesa, 1882* (Lisbon: Imprensa Nacional, 1890), 238.

56.
‘Relatório dos Serviços de Saúde do Concelho de Bissau de 1890’, 6 March 1891; AHU, Secretaria de Estado da Marinha e do Ultramar (SEMU), Direccção Geral do Ultramar, Repartição da Saúde, Serviços de Saúde, Cx. 2: Guiné, 1888–99.

57.
Carlos Pereira, *La Guinée Portugaise: subsides pour son étude* (Lisbon: A Editora Limitada, 1914), 27.

58.
‘Regulamento da Polícia da Prostituição’, Bolama, 10 August 1915, *Boletim Oficial da Guiné Portuguesa*, 33, 14 August 1915.

59.
Ndao, *op. cit.* (note 36), 14.

60.
M. Nogue, ‘La syphilis chez les noirs au Sénégal: la lutte contre la syphilis à Dakar’, *Revista Médica de Angola*, III, 4 (1923),179–99.

61.

*A Voz da Guiné*, 1, 6 April 1922, 4, 18 May 1922.

62.
Luiz António de Carvalho Viegas, ‘Relatório do Governador da Colónia da Guiné de 1936’, Bolama, 28 July 1937, 107; AHU, MU, ISAU, 166.

63.
Sant’Ana Barreto, ‘Climatologia e Nosographia da Guiné Portuguesa’, in *Anuário da Guiné Portuguesa* (Bolama: Imprensa Nacional, 1925), 149.

64.
José Vitorino Pinto, *Relatório sobre uma visita sanitária feita aos Bijagós* (Bolama: Imprensa Nacional, 1928), 3.

65.
Luiz António de Carvalho Viegas, ‘Relatório do Governo da Guiné, 1935’, Bolama, 28 June 1936, 184; AHU, MU, DGCOcd 400.

66.
Dube, *op. cit.* (note 13), 104–05.

67.
Leite de Noronha, ‘Relatório dos Serviços de Saúde, Bolama, 10 September 1937’, in Luiz António de Carvalho Viegas, ‘Relatório do governo da Colónia da Guiné, Trimestre de 1937’; AHU, ISAC, 545.

68.
Luiz António de Carvalho Viegas, ‘Relatório do Governo da Colónia da Guiné, 1st Quarter 1937’, Bolama 29 July 1937; AHU, ISAC, 545.

69.
Lopo Vaz Sampayo de Mello, ‘O eterno feminino nas colónias’, *O Mundo Português*, 3, (1936), 176.

70.
Rui Randolfo Roncon, ‘Relatório Circunscrição Sanitária de Bafatá’, in Fernando José Oliveira de Montalvão e Silva, ‘Relatório Anual dos Serviços de Saúde, Bissau’, 30 June 1942, 68; AHU, MU, DGSA, RSH-004, Cx. 6.

71.
See A. Vadier, ‘Rapport sur la Situation Politique en Guinée Française en 1933; Archives Nationales d’Outre-Mer (ANOM), FM, 1AffPol, c. 982’, GP-1933-34; Conseil General de la Guinée-Française, ‘Compte rendu, Session Extraordinaire, Conakry, 25 April 1947, ANOM, 1AffPol, c. 2164’; C. Becker and R. Collignon, ‘Politiques démographiques et sanitaires face aux “maladies sexuellement transmises” en Afrique de l’Ouest: perspective historique’, in C. Becker *et al.*, *op. cit.* (note 23),133–49: 139; Ndao, *op. cit.* (note 36), 4–6.

72.
Luiz António de Carvalho Viegas, ‘Relatório do Governo da Colónia da Guiné de 1938’, Bolama, 1939,9; AHU, MU, ISAC, 544.

73.
João Nunes de Oliveira, ‘Inspecção Geral aos Serviços Centrais da Colónia da Guiné’, Lisbon, 1 August 1942, 122; AHU, MU, ISAU (Inspecção Superior da Administração Ultramarina).

74.
Francisco Borja Santos, ‘Relatório do Curador Geral do ano de 1942’, Bissau, 20 October 1943, 27; AHU, MU, ISAU 1725; Roncon, *op. cit.* (note 74), 69–70.

75.
José Salvação Barreto, ‘Relatório da Inspecção Ordinária aos Serviços Adminstrativos da Colónia da Guine, 1936–37’, Lisbon, 18 March 1938, 3; AHU, MU, ISAU. The first village infirmaries with a section specialised in yaws were built in the mid-1930s.

76.
Francisco Borja Santos, ‘Relatório do Curador Geral dos Serviços de Negócios Indígenas, ano de 1942’, Bissau, 20 October 1943; AHU, MU, ISAU 1725.

77.
José d’Assunção Barreto, ‘Recommendation’, Lisbon, 21 November 1940; AHU, MU, ISAU, 1725.

78.
Conselho do Governo da Guiné Portuguesa, ‘Minutes’, Bolama, 2 March 1944; AHU, MU, DGAPC, 581.

79.
Nunes de Oliveira, *op. cit.* (note 73), 122. The *mestras* in Chistianised strata in towns such as Bissau, to whom rural families entrusted their young daughters for an urban education, were viewed as the main culprits behind the organisation of prostitution rings.

80.

*Conferência dos Administradores: Actas das Sessões* (Bolama: Imprensa Nacional, 1943), 95–97.

81.
Fernando Carlos de Magalhães Mendonça Pimentel, Director Colonial Health Services, ‘Relatório Anual dos Serviços de Saúde de 1945’, Bissau, 31 March 1946; AHU, MU, DGSA, RSH-004, Cx. 6.

82.
Ndao, *op. cit.* (note 36), 5.

83.
Pimentel, *op. cit.* (note 81), 5.

84.
Mário Ludgero Viegas, District Health Officer Farim, in Silva, *op. cit.* (note 70), 45.

85.
Fernando José Oliveira de Montalvão e Silva, Bolama, 27 November 1942, in *op. cit.* (note 80), 81.

86.
António Carreira, Organização social e económica dos povos da Guiné Portuguesa, Boletim Cultural da Guiné Portuguesa, XVI, 64 (1961), 641–736: 667; António de Almeida, ‘Da medicina gentílica dos Bijagós’, in Ministério das Colónias, *Conferência Internacional dos Africanistas Ocidentais*, vol. V (Lisbon: Junta de Investigações Coloniais, 1952), 243–92: 271–722.

87.
José F. Tavares Fortuna, ‘Monografia das Delegacias de Saúde de Bolama e de Bubaque, Bolama’, December 1958; AHU, MU, DGSA, 3215, 25.

88.
António Carreira, ‘Taxas regionais de natalidade e mortalidade infantil’, *Boletim Cultural da Guiné Portuguesa*, VII, 27 1952, 533–44.

89.
Similar trends for the same period are recorded for Senegal; Ndao, *op. cit.* (note 36), 8–9.

90.
Luis Espírito Santo Graça, ‘Relatório dos Serviços da Delegacia de Catió’, in Rui Randolfo Roncon, Relatório dos Serviços de Saúde e Higiene (1950)’, Bissau, 13 September 1951; AHU, MU, DGSA, RMS-004, Cx. 6., p. 100; Tavares Fortuna, *op. cit.* (note 87), 15.

91.
Ruy Álvaro Vieira, ‘Relatório do Delegado de Saúde de Bolama’, 1 March 1949, p. 21; AHU, MU, DGSA, RSH-004, Cx. 6.

92.
Fernando Leite de Noronha, ‘Relatório da Delegacia de Bafatá’, 15 March 1951, 62; AHU, MU, DGSA, RSH-004, Cx. 6.

93.
Júlio Cesar Queirós Valente de Almeida, ‘Resposta ao questionário, Bafatá’, 6 December 1958, pp. 3–5; AHU, MU, DGSA 3215, 25.

94.
WHO Expert Committee on venereal diseases, ‘Report by director general, EB2/7’, 19 August 1947; WHO Expert Committee on Venereal Infections, ‘Report of Third Session’, *British Journal of Venereal Diseases*, 26, 2 (1950), 77.88; WHO Expert Committee on Venereal Infections and Treponematoses, ‘4th Report’ (Geneva: WHO, 1953.

95.
Havik, *op. cit.* (note 14), 656–57.

96.
Hostains, French Consul, to French Ministry of Colonial Affairs, Bissau, 10 June 1922; ANOM, FM, 1AffPol, c. 1291, Guinée Portugaise, 1907–36.

97.
Barthelémy, *op. cit.* (note 32), 125. On the training and practise of midwives in AOF, see Jane Turittin, ‘Colonial midwives and modernizing childbirth in French West Africa’, in Jean Allman, Susan Geiger and Nakanyike Musisi (eds), *Women in African Colonial Histories* (Bloomington: Indiana University Press, 2002), 71–91.

98.
Mor Ndao, ‘Enfance et ordre Colonial: la politique sanitaire au Sénégal, discours et realité 1930–60)’, *Révue Sénegalaise d’Histoire*, 2005, 1–13, https://halshs.archives-ouvertes.fr/halshs-01304675(accessed 17 September 2016); Barthélémy, *op. cit.* (note 32), 126–27; Turittin, *ibid.*, 71–72.

99.
Amponsah, *op. cit.* (note 33), 96–98.

100.
Van Tol, *op. cit.* (note 33), 112–13.

101.
Almeida, *op. cit.* (note 46), 11.

102.
Fundo de Assistência Médica Indígena/Fundo de Assistência Agrícola e Zootécnica, Decree 728/818, Bolama, 20 December 1932/30 December 1933, *Boletim Oficial da Guiné Portuguesa*, 51, 18 December 1932.

103.
Ndao, *op. cit.* (note 98), 3.

104.
Fundo de Assistência Médica Indígena, *op. cit.* (note 102).

105.

*Ibid*, 4–15. On village infirmaries, see Havik, *op. cit.* (note 45), 22–5.

106.
Pedro de Araújo, ‘Relatório dos Serviços Missionários referente ao ano de 1933–34’, Bula, 1 June 1935; AHU, MU, DGENS, 2344.

107.
Família indígena, in: Luiz António Carvalho Viegas, *Guiné Portuguesa*, vol. III (Lisbon: Severo Freitas e Mega Cia, 1940), 219–391.

108.
Viegas, Relatório do Governo, p. 162; AHU, MU, Gabinete do Ministro das Colónias (GM) 2879 (note 68).

109.
Carvalho Viegas, *op. cit.* (note 68), 177.

110.
Araújo, *op. cit.* (note 106). Catholic missionaries complained that they were not regarded as valid health care providers, despite receiving free medicines from the Fund for their mobile services in the interior; Augusto Pereira Brandão, director of Colonial Health Services, confidential, Bissau, 25 June 1935 to Governor; AHU, MU, DGENS 2344.

111.
Joaquim Dias, ‘Relatório Anual das Missões Católicas Portuguesas da Colónia da Guiné (1940)’, Bolama, 30 April 1941; AHU, MU, DGENS 2344.

112.
José N. Nunes de Oliveira, ‘Relatório da Inspecção-Geral aos Serviços Centrais da Colónia da Guiné (1942)’, Lisbon, 1 August 1942.

113.
Luiz António de Carvalho Viegas, ‘Relatório do Governo de 1939’, Bolama, 25 October 1940, 15; AHU, MU, GM 2940.

114.
Barreto, *op. cit.* (note 75), 32.

115.
Ricardo Vaz Monteiro, ‘Relatório do Governo, 1943’, Bissau, 30 August 1944, 36–9; AHU, MU, ISAU 2246.

116.
Augusto Pereira Brandão, ‘Relatório dos Serviços de Saúde da Colónia da Guiné, 1936’, Bissau, 31 March 1937; Luiz António de Carvalho Viegas, Relatório do Trimestre (1937), Bolama, 29 July 1937; AHU, MU, ISAC 545.

117.
Decree no. 45541, *op. cit.* (note 44); art. 3, Decree 165, Bissau, 30 December 1946 regulating health services in PG.

118.
Maurice Blanchard, ‘l’Oeuvre Sanitaire de la France en AOF (partie administrative)’, Clermont-Ferrand, 31 December 1940, 46–56; ANOM, 1AffPol, 3240.

119.
António de Almeida, ‘Relatório da Inspecção Extraordinária da Guiné Portuguesa’, May 1949; AHU, ISAU.

120.
Fernando Carlos de Magalhães Mendonça, ‘Relatório Anual dos Serviços de Saúde e Higiene’ (1948), 3–12; AHU, MU, DGSA, RSH-004, Cx.6.

121.
M.A.M. Sarmento Rodrigues, ‘Relatório da Conferência dos Administradores da Colónia da Guine, 1946’, Bissau, 29 July 1946, 18; AHU, MU, ISAU 1731.

122.
H. Bogaers, ‘French consul in Bissau’, 8 May 1947; MAE, K-Afrique, Afrique Levant, 1944–52, M. 24, Possessions Portugaises, Guinée Portugaise.

123.
Fernando Carlos de Magalhães Mendonça, ‘Relatório Anual dos Serviços de Saúde e Higiene’ (1945), 2; AHU, MU, DGSA, RSH-004, Cx. 6. In the 1920s a school for indigenous nurses operated in Bolama, but was discontinued for lack of funds and personnel.

124.
Roncon, *op. cit.* (note 90), 19.

125.
Júlio Cesar Queirós Valente de Almeida, ‘Relatório dos Serviços de Saúde da Delegacia de Teixeira Pinto, 31 December 1950’, in Roncon, *op. cit.* (note 68), 30.

126.
Almeida, *ibid.*, 10.

127.

*Op. cit.* (note 85), 74.

128.

*Ibid.*, 88–90.

129.
Carlos Eugénio Bessa Victor, ‘Relatório dos Serviços de Saúde da Delegacia de Mansoa, 31 December 1950’, in Roncon, *op. cit.* (note 90).

130.
Ricardo Vaz Monteiro, ‘Relatório do Governo da Guiné (1943)’, Bissau, 30 August 1944, 37; AHU, MU, ISAU 2246.

131.
Tavares Fortuna, *op. cit.* (note 87); José Tavares Fortuna, Personal communication, 21 March 2017.

132.
Augusto Pereira Brandão, ‘Relatório dos Serviços de Saúde, 31 December 1936’, in Luiz António de Carvalho Viegas, ‘Relatório da Colónia da Guiné (1936)’, Bolama, 28 June 1937, 106; AHU, MU, ISAU 1667.

133.
Tavares Fortuna, *op. cit.* (note 87), 42.

134.
José Tavares Fortuna, ‘Os médicos do mato africano’, in Isabel Silva Ribeiro (ed.), *África, Médicos e Memórias* (Lisbon: By the Book, 2016), 170–86.

135.
Carlos Henrique Jones da Silveira, ‘Relatório da Inspecção Administrativa Ordinária na Colónia da Guiné, 1950–51’, Bolama, 30 June 1951, 383; AHU, MU, ISAU.

136.
Júlio Cesar Queirós Valente de Almeida, ‘Reposta ao questionário, Nova Lamego (Gabú)’, 6 December 1958; AHU, MU, DGSA 3215.

137.
Dispatch 81/1/1, Luanda, 7 April 1958, M. da Cruz Alvura to DGAPC, Lisbon; AHU, UM, DGSA 3213.

138.
Havik, *op. cit.* (note 45), 49.

139.
See for example, Dispatch RC6/PRB 56-57, AFRO, Brazzaville, 22 February 1956, signed Francisco J.C. Cambournac to Ministry of Foreign Affairs (MNE), Lisbon; AHU, MU, DGAPC, Maço 55, Pasta 22.

140.
Dispatch, Serviços de Saúde e Higiene, Lisbon, 17 August 1955, Santana Marques, to Francisco J.C. Cambournac, AHU, MU, DGAPC, Maço 55, Pasta 20.

141.
Dispatch, BRA, Afr.S.17/Afr.CL., Brazzaville, 21 March 1955, signed Francisco J.C. Cambournac to MNE, Lisbon; and Telegram no. 100, Governor PG to Ministry Colonial Affairs, Bissau, 15 June 1955; AHU, MU, DGAPC, Maço 55, Pasta 23C-2.

142.
Rui Roncon, ‘Relatório Circunscrição Sanitária de Canchungo’, in Pimentel, *op. cit.* (note 81), 38.

143.
Manuel Bento Gonçalves Ferreira, ‘Relatório da Inspeccão Ordinária a Província da Guiné (1959/60)’, 27; AHU, MU, ISAU.

144.

*Ibid.*, 27.

145.
José Tavares Fortuna, personal communication, 11 June 2018.

146.
José Tavares Fortuna, personal communication, 28 February 2017.

