# Public Health, Social Medicine and Disease Control: Medical Services, Maternal Care and Sexually Transmitted Diseases in Former Portuguese West Africa (1920–63) – ADDENDUM

**DOI:** 10.1017/mdh.2018.80

**Published:** 2019-01

**Authors:** Philip J. Havik

doi:10.1017/mdh.2018.44, Published online by Cambridge University Press, 07 September 2018

The above article was originally submitted to Cambridge University Press with some missing funding information. This information is as follows:

The author would like to acknowledge the financial support of the Fundacão para a Ciência e Tecnologia (FCT) in Lisbon (Research Project IF/01130/2013/CP1165/CT0002), and thank anonymous referees for their insightful comments and Alexander Metcalf for his diligence.^1^

## Footnotes

1.

P.J. Havik , ‘Public Health, Social Medicine and Disease Control: Medical Services, Maternal Care and Sexually Transmitted Diseases in Former Portuguese West Africa (1920–63)’, Medical History, 62 (2018), 485–506.3019178410.1017/mdh.2018.44PMC6158648

